# Emotional-Behavioral Functioning, Maternal Psychopathologic Risk and Quality of Mother–Child Feeding Interactions in Children with Avoidant/Restrictive Food Intake Disorder

**DOI:** 10.3390/ijerph17113811

**Published:** 2020-05-27

**Authors:** Luca Cerniglia, Eleonora Marzilli, Silvia Cimino

**Affiliations:** 1 Uninettuno and Faculty of Psychology, International Telematic University, 00186 Rome, Italy; 2 Department of Dynamic and Clinical Psychology, Sapienza University of Rome, 00186 Rome, Italy; eleonora.marzilli@uniroma1.it (E.M.); silvia.cimino@uniroma1.it (S.C.)

**Keywords:** avoidant/restrictive food intake disorder, diagnostic subtypes, mother–child feeding interactions, maternal psychopathologic risk, children emotional-behavioral functioning

## Abstract

The literature on food intake disorder (ARFID) in early childhood has evidenced psychopathologic difficulties in both children and their mothers and a poor quality of feeding interactions. Only a few studies have focused on three different ARFID subtypes: irritable/impulsive (I/I), sensory food aversions (SFA) and post traumatic feeding disorder (PTFD). The aim of this study was to explore possible differences between the three groups in children’s emotional-behavioral functioning, maternal psychopathologic risk and the quality of mother–child feeding interactions, comparing these clinical groups with a control group. The sample consisted of 100 child–mother dyads, of which 23 children with I/I, 25 children with SFA, 27 children with PTFD and 27 children with no diagnosis. The mothers primarily filled out questionnaires assessing their psychopathologic symptoms and children’s emotional-behavioral functioning. Then, all dyads were videotaped during a main meal. Results revealed significant differences between the study groups in relation to children’s emotional–adaptive functioning, mothers’ psychological profile and mother–child interactions during feeding. These findings are relevant for the development of target intervention programs to treat specific ARFID disorders.

## 1. Introduction

The Fifth Edition of the Diagnostic and Statistical Manual of Mental Disorders (DSM-5) [1] has recently included the new diagnostic category of avoidant/restrictive food intake disorder (ARFID) in the section of feeding and eating disorders, updating the previous, more broadly defined, clinical label of feeding disorder of infancy or early childhood that was present in the DSM-fourth edition-text revision (DSM-IV-TR) [2]. In particular, feeding disorders criteria of the fourth edition had been criticized as of low clinical utility and for an excessive attention to weigh loss (that is not always present in patients, especially in young children) [3], and had been therefore complementary integrated by the use of the zero-to-three (DC: 0–3) classification [4]. On the other hand, the current DSM-5 approach to feeding disorders in children has been defined an important step forward in the field, which can improve both diagnostic practice and clinical intervention [5].

Children with ARFID show difficulties in feeding and eating, with inadequate food intake that can be associated with insufficient ingestion of required nutrients and energy intake. Even if they do not show a significant weight loss (e.g., because of the use of nutritional supplements, often supplied by parents), individuals with ARFID manifest impaired social and psychological functioning [1]. Moreover, disordered feeding and eating behavior must not be explained by other medical conditions or psychiatric problems.

Very importantly, it has been suggested that ARFID encompasses different clinical manifestations [6,7]. One possibility is that subjects display a lack of interest in food or eating (young children, in particular, may seem to have a reduced response to physiological hunger cues and may not protest and request to be fed even after many hours from the last meal). Other patients (especially older children) may show a high selectivity and avoidance of certain foods, based on their smell, texture or appearance, accepting to eat only a restricted variety of foods. Yet another group of individuals may display food avoidance subsequent to previous distressing experiences involving oro-pharyngeal and gastrointestinal tract and may restrict and/or avoid their feeding and/or eating to prevent anticipated choking or vomiting. The characteristics of these clinical manifestations had been described by Chatoor [8] in accordance with the DC:0–3 classification [respectively with the clinical labels of: infantile anorexia (IA), sensory food aversion (SFA) and Feeding Disorder associated with insults to the gastrointestinal tract, also named post-traumatic feeding disorder (PTFD)]. This classification has recently been replaced by the new zero-to-five taxonomy, based on important recommendations by worldwide experts in this field [9]. Moreover, the presence of another ARFID subtype [irritable/impulsive (I/I)] has been posited. In fact, some children with ARFID seem to have less self-control [10] or appear irritable and difficult to console during meals [11,12]. Recent evidence suggested the presence of significant association between food avoidant behaviors and impulsivity symptoms in preschool children [13] and the insufficient nutritional intake can further aggravate these associated characteristics [11].

However, it has not been explored whether these different manifestations are associated to specific children’s psychological profiles. In general, a large bulk of research has demonstrated the association of feeding disorders with children’s difficult temperament and increased levels of physiological arousal [14,15,16,17,18], also showing higher internalizing and externalizing symptoms in these children [19,20]. Although difficulties in emotion and behavior regulation have been proposed as associated with ARFID symptomatology, anorexia nervosa and bulimia in adolescents and adults, much fewer studies have focused on dysregulation in children with ARFID [21]. Importantly, no study to our knowledge has so far evaluated dysregulation symptoms as associated with the different ARFID subtypes. Yet, the presence of an impaired dysregulation profile (DP) in children has recently been posited as a significant predictor for major psychopathology in later life, deserving particular attention and being potentially very useful to inform diagnostic and intervention programs.

Using the developmental psychopathology framework, some studies have shown that mothers of children with feeding disorders are often characterized by psychopathologic symptoms (especially anxiety and depression) [22,23,24,25]. However, no study to our best knowledge focused on mothers of children with ARFID, specifically considering the above-described sub-types. Interestingly, several studies have demonstrated that the feeding interactions between children with feeding disorders and their mothers are frequently characterized by low quality of interactional exchanges, especially during feeding [26,27,28]. Very few studies, however, evaluated this aspect differentially for the ARFID diagnostic sub-types [29,30,31,32,33]. Therefore, there is an urgent need of research to accumulate data and results useful for the implementing of diagnostic and intervention programs specific for children with ARFID.

Through a consecutive sampling approach, the present study has aimed to, at least partially, fill this gap by recruiting a clinical sample of children with ARFID and their mothers (divided into the three sub-groups based on clinical presentations as suggested by Norris and colleagues [32]: I/I subtype, SFA subtype, PTFD subtype) and a control group matched by sociodemographic characteristics. Our specific objectives were to: (1) verify a possible significant difference on children’s emotional-behavioral functioning between the four groups, assessing internalizing, externalizing and dysregulation symptoms; (2) verify possible differences in maternal psychopathologic risk between the study groups; (3) evaluate the quality of mother-child interactions during feeding in the four groups. As no previous research has focused on these specific objectives, no hypotheses were formulated and the study had an explorative nature.

## 2. Materials and Methods

### 2.1. Participants

The sample consisted in one hundred and sixty-one mother–child dyads recruited through mental health clinics in central Italy. Mothers were contacted by psychologists who explained the aims of the study and written informed consent was obtained. The study was approved by the Ethical Committee of the Psychology Faculty at the International Telematic University Uninettuno (n. 2018/3), in accordance with the Declaration of Helsinki. The diagnosis of the three ARFID subtypes was made independently by two clinicians (Cohen’s k = 0.80), based on the criteria of the DSM-5 [1] for Feeding and Eating Disorders, of the DC:03-R criteria [4] and on clinical presentations suggested by Norris and colleagues [32]. Inclusion criteria for the clinical groups were a children ARFID diagnosis, with specific reference to three sub-types (I/I, SFA and PTFD), without a comorbid disorder. We excluded families in which children and/or mothers were following a pharmacological or psychological treatment (N = 8); families in which children had a comorbid feeding disorder (N = 21) or in which mother and/or child had physical, cognitive or neurological impairments (N = 4); families in which mothers did not complete all the questionnaires (N = 15); and families who refused to participate in the study (N = 13).

The final sample included 100 children from 24 to 36 months (M = 30 months, SD = 3.07; 50% females) and their mothers (M = 31 years, SD = 2.4). On the basis of the children’s diagnoses, the sample was divided into four subgroups: (1) I/I group, composed by mother–child dyads in which child were diagnosed with ARFID I/I subtype (N = 23); (2) SFA group, composed by mother–child dyads in which child were diagnosed with ARFID SFA subtype (N = 25); (3) PTFD group, composed by mother–child dyads in which child were diagnosed with ARFID PTFD subtype (N = 25); (4) NC group, composed by families in which child received no diagnosis (N = 27). Most mothers had high school (81.4%) or university (27.6%) education and only 2.3% of mothers had only middle school education. The vast majority of mothers had average socioeconomic status (91% had an average income of 25,000–30,000 Euros per year).

### 2.2. Procedure

We have randomly selected the order of administration of the measures (described below). In particular, the mothers filled out the child behavior checklist (CBCL 1.5–5) [34] for the assessment of children’s emotional-behavioral functioning and the Symptom Checklist-90-Revised (SCL-90-R) [35] for the assessment of their psychopathologic symptoms. These tools were chosen because they are very widely used in international research and proved their validity to capture a wide range of psychopathologic difficulties that can be experienced by mothers and children [17,36,37]. Moreover, mother–child feeding interactions were videotapes during a main meal (20-min videos) at the family home, based on a validated procedure [38,39] and in line with previous studies [25,40]. The mother–child feeding exchanges were then coded by two trained independent raters (Cohen’s k = 82). We have chosen this tool because it is the only observational procedure for the assessment of mother–child feeding interactions validated for the Italian population.

### 2.3. Measures

The CBCL 1.5–5 [34,41] is a 99-item informant–report questionnaire for the assessment of emotional/behavioral problems of child during the past six months. Parents are asked to rate the items on a three-point Likert scale ranging from 0 (not true) to 2 (very true or often true) and they are grouped on the following syndrome scales: emotionally reactive, anxious/depressed, somatic complaints, withdrawn, attention problems, aggressive behavior and sleep problems. In turn, these subscales are grouped into two broad-band scales: internalizing problems (which combines the items from the emotionally reactive, anxious/depressed, somatic complaints, withdrawn scores) and externalizing problems (comprised of items attention problems and aggressive behavior). For the CBCL DP measurement, we summed the items of the syndrome scales anxious/depressed, attention problems and aggressive behavior [42]. For the statistical analyses we used the raw scores.

The SCL-90-R [35] is a 90-item self-report questionnaire aimed at measuring psychological symptoms and psychological distress. Its nine primary dimensions are: somatization, obsessive-compulsivity, interpersonal sensitivity, depression, anxiety, hostility, phobic anxiety, paranoid ideation and psychoticism. Moreover, it provides a global severity index (GSI) that is used to the severity and degree of psychological distress. The Italian validation [43] showed a good internal consistency in adolescents and adults (Cronbach’s α = 0.70–0.96), with a clinical cutoff score of 1.

The Scala di Valutazione dell’Interazione Alimentare (SVIA) [39] is the Italian adaptation of the Feeding Scale [38], which can be used to evaluate interactive behavior in children with 1–36 months of age. The Italian version has 41 items—rated on a four-point Likert Scale—through which identify normal and/or risky mother–child feeding interactions. The items are distributed among four subscales: affective state of the mother (index of the parents’ affective states); Interactive conflict (index of interactions characterized by conflictual, non-collaborative and non-empathetic communication); Food-refusal behaviors of the child (habits associated with challenged status regulation during meals and with limited food consumption); and Affective state of the dyad (index of the extent to which the infant’s feeding patterns are or are not, the result of an interactive regulation to which maternal contribute). Higher scores in each scale refer to greater difficulties. Moreover, it provides a Total score (that consisted in the sum of the four subscales), used as a general measure of the quality of mother–child feeding interaction. Scores over 50 are indices of clinical scores [44,45]. The SVIA showed a good reliability in terms of internal consistency (Cronbach’s α = 0.79–0.96).

### 2.4. Statistical Analyses

Preliminary analyses were performed using descriptive statistics (frequencies, percentages, mean scores and the reliability of the measures). To verify a possible significant difference on children’s emotional-behavioral functioning between the four groups (aim 1), one-way analysis of variance (ANOVA) was conducted considering the mean scores of the CBCL 1.5–5 Syndrome Scales and of the CBCL DP. Since the Levene’s test showed the presence of a non-homogeneity of variance (Levene test, *p* < 0.05), except for anxious/depressed and attention problems scales, Welch’s test was conducted for all other scales. Tukey’s post hoc tests (used for scores of anxious/depressed and attention problems scales) and Dunnett T3 post hoc test (used for other scales) were used to identify differences between the sample means. Then, in order to verify possible differences in maternal psychopathologic risk between the study groups (aim 2), ANOVAs were carried out, with the group as the independent variable and each of the SCL-90/R subscales as the dependent variable. The Levene’s test was significant for all scales. Consequently, we used Welch’s test. Multiple post hoc comparisons were conducted using the Dunnett post hoc test. Finally, the differences between the study groups on the observational scale during feeding (aim 3) were examined using ANOVAs with the group as the independent variable and each of the four subscales and the Total Scale of the SVIA as dependent variables. We conducted Welch’s tests, because the Levene’s test was significant for all subscales. Dunnett’s T3 multiple comparisons post hoc tests were used to analyze group differences. For all analyses, we set the alpha level at 0.05. All analyses were performed using IBM SPSS Statistics software, Version 25.0 [46].

## 3. Results

### 3.1. Children’ s Emotional-Behavioral Functioning in the Four Groups

The results showed that the four groups were significantly different on scores of emotionally reactive, anxious/depressed, somatic complaints, withdrawn, sleep problems, attention problems, aggressive behavior, internalizing problems, externalizing problems and DP (Table 1).

Post hoc tests showed that children with no diagnosis had lower scores than other groups in scales of emotional reactivity, depression, sleep, aggression, internalizing, externalizing problems and DP and lower levels of somatic compliance, withdraw and attention problems scales than children with I/I and PTFD diagnosis. Children with I/I diagnosis had higher scores than other groups in the scores of anxious/depressed, attention problems and internalizing problems scales. Moreover, children of I/I group reported higher scores than children of SFA group on somatic compliance, withdraw, sleep problems, aggressive problems, externalizing problems scales and DP. Children with SFA diagnosis showed higher scores on emotional reactivity scale than other groups and lower scores on the DP compared to other clinical groups. Finally, children with PTFD had higher levels of aggression and externalizing problems than children of other groups. In addition, children with PTFD, compared to children with SFA, had higher levels on somatic compliance, withdraw and DP (Table 2).

### 3.2. Mothers’ Psychopathologic Risk in the Four Groups

The results showed the presence of significant differences between the four groups in maternal scores of SCL-90-R on the subscales of somatization, obsessive compulsion, depression, interpersonal sensitivity, anxiety, hostility, phobic anxiety, paranoid ideation, psychoticism and GSI (Table 3).

Dunnett T3 post hoc tests showed that mothers of children with no diagnosis reported lower scores than other groups in all subscales of SCL-90/R considered. Mothers of children with I/I diagnosis obtained scores over the clinical cutoff in all SCL-90/R dimensions and GSI. Compared to mothers of other groups, they had higher score on GSI, as well as higher scores on the subscales of somatization, interpersonal sensitivity, depression, hostility, phobic anxiety, paranoid ideation and psychoticism. Moreover, they reported higher levels of obsessive compulsion and anxiety than mothers of children with SFA diagnosis. Mothers of children of SFA group reported lower scores on all considered subscales compared to mothers of children with a diagnosis. Finally, mothers with children with PTFD diagnosis showed higher scores of obsessive compulsion and anxiety compared to other groups. Moreover, they obtained scores over the clinical cutoff in all SCL-90/R dimensions and GSI (Table 4).

### 3.3. Quality of Mother-Child Interactions during Feeding in the Four Groups

The results showed the presence of significant differences between groups in the scores of affective state of the mother, interactional conflict, food refusal of the child, affective state of the Dyad and Total Score of SVIA (Table 5).

Dunnett T3 post hoc test showed that NC group reported lower scores in all subscale and in the Total score of SVIA compared to clinical groups. The I/I group showed higher scores in all subscale and in the Total score of SVIA compared to other groups. The scores of all subscale and total scale of SVIA exceeded the clinical range cutoff. The dyads of the SFA group had all scores under the clinical cutoff. Moreover, the PTFD group had higher scores than SFA group in all subscales and in the Total score of SVIA and the dyads of this group had all score over the clinical cutoff (Table 6).

## 4. Discussion

The goal of the study was to investigate emotional-behavioral functioning in a sample of preschooler children with three specific sub-types of ARFID diagnosis (I/I, SFA and PTFD), psychopathologic risk of their mothers, and the quality of mother–child feeding interactions, comparing these clinical groups with a control group. We based on transactional model, which considered the bidirectional interplay between children’s and mothers’ characteristics on the onset of developmental psychopathology, that in turn may lead to a poor quality of dyadic interactions during feeding [47]. International literature in this field has shown that children with early feeding disorders are at risk of a wide range of psychopathologic difficulties, both in internalizing and externalizing areas [19,20,48,49] and regulatory problems [50,51]. Moreover, mothers of children with a feeding disorder also manifested the presence of psychopathologic symptoms [52], and a poor quality of feeding interactions with their children [17,24,26,53]. However, to date, there is a dearth of studies focused on children with different subtypes of ARFID, and no study has considered the possible role played by children’ CBCL DP, a clinical condition characterized by a poor self-regulation and a co-occurrence of internalizing and externalizing problems [54].

Overall, the results of the study have evidenced specific features associated with each ARFID subtypes. In particular, children with I/I diagnosis showed higher anxiety/depressive symptoms, attentive problems and internalizing problems than children of other groups. Their mothers, compared to other groups, had the highest score on GSI and higher scores on the dimensions of somatization, interpersonal sensitivity, depression, hostility, phobic anxiety, paranoid ideation and psychoticism. Moreover, they obtained scores over the clinical cutoff in all SCL-90/R dimensions, especially depression. Finally, the dyads of the I/I group, showed higher scores in all subscale and in the Total score of SVIA compared to other groups (indicative of the poorest quality of dyadic feeding interactions), with scores over the clinical range cutoff for all dimensions of SVIA. This is the first study that has explored emotional-behavioral functioning, maternal psychopathologic risk and the quality of mother–child feeding interactions among children with I/I ARFID subtype. However, previous literature has evidenced that food refusal in early childhood may be associated with the presence of hyperactive behavior overtime [55,56] and that children with internalizing and attention problems (as those diagnosed I/I were) and/or with feeding problems, often have mothers with psychopathologic difficulties, especially in the depression area [57,58,59]. Moreover, although no other study have explored the quality of feeding interaction among these dyads, our results are in accordance with previous findings on children with a feeding disorder [17,23,25,29,60]. These studies have evidenced exchanges characterized by low dyadic reciprocity, interactional conflict and negative affect in both mothers and their children.

Another children’s ARFID subtype that was examined was SFA. These children had higher scores on Emotional reactivity scale, but lower score on the CBCL DP than other clinical groups. Moreover, compared to children of I/I and PTFD groups, they had lower scores on somatic compliance and withdraw and lower levels of sleep problems, aggressive problems and externalizing problems than children with I/I diagnosis. As expected, mothers of this group had higher psychopathological risk than mothers of NC group. However, compared to mothers of other clinical groups, they reported lower scores on all considered SCL-90/R subscales that were under the clinical cutoff. Moreover, the dyads of the SFA group had all scores of SVIA under the clinical cutoff and showed the better quality of feeding interactions among clinical groups. In particular, although these dyads had higher scores in all subscales and in the Total score of SVIA compared to NC group, they reported lower scores in the same SVIA dimension than other ARFID subtype groups. These findings are consistent with previous studies. In particular, the study by Lucarelli and coll. [17] showed that children with SFA manifested higher emotional reactivity problems than other ARFID subtypes (i.e., IA, PTFD), but lower emotional-behavioral problems in the other dimensions. Moreover, they reported no clinical symptomatology in mothers of children with SFA and a lower psychopathologic profile respect to mothers of children with other feeding disorders [17]. Researchers in the field of developmental psychopathology framework have suggested that both child and maternal characteristics may be significant predictors of the quality of mother–child interactions during early childhood [23,24,25]. Consequently, the lower level of psychopathologic difficulties found in both children and mothers of SFA group seems to have been reflected in the better quality of mother–child feeding interactions, in accordance with previous studies [17].

Finally, children with PTFD had more difficulties in externalizing area, showing higher levels of aggression and externalizing problems than children of other groups. Moreover, although there was no significant difference compared to children with I/I, they showed the highest DP scores compared to other groups. As regards mothers of children with PTFD diagnosis, they showed higher scores of obsessive compulsion and anxiety than other groups, and they obtained scores over the clinical cutoff in all SCL-90/R scales and GSI. Considering mother–child feeding interactions, these dyads showed all SVIA scores over the clinical cutoff and higher scores than NC and SFA groups, but lower scores than I/I group in all SVIA dimensions. The link between PTFD diagnosis and children’s externalizing problems, maternal psychopathologic difficulties (especially in the obsessive compulsion and anxiety areas), as well as a poor quality of dyadic feeding interactions is in line with previous studies in this field [17]. As suggested by some authors [61,62], the presence of child’s feeding difficulties may be a psychological stressor for mothers that can spillover into the quality of interactions with their children. However, this study is the first that also had underlined the presence of dysregulation problems among these children. As showed by other studies on early traumatic events, a child exposed to a traumatic experience at an early age is at greater risk of manifest dysregulation problems [63,64,65]. Our study suggested that also a food-related trauma may lead to subsequent emotional and behavioral self-regulation difficulties.

### 4.1. Limitations and Strengths

Our study has some limitations. In fact, many studies have highlighted the kay role assumed also by paternal psychological functioning [66,67,68] and the quality of father-children relationship [26,45]—especially in the context of child feeding practices [69]—in shaping children’s emotional-behavioral development and feeding problems [70]. However, we did not evaluate the role played by fathers, in terms of their psychopathologic risk and the quality of father-child feeding interactions, which it will be useful to explore in future research. Moreover, we assessed the mother’s psychopathologic risk using self-report measure, and future studies should assess this aspect through clinical interviews. Previous studies have also shown that mothers of children with eating disorders often show symptoms of eating disorders, highlighting the presence of possible intergenerational transmission of the disorder [71,72]. However, we have not assessed the possible occurrence of eating disorders in mothers. Therefore, further studies should consider the possible influence of the-on-the occurrence of ARFID in children. Another limitation of the study is related to the generalizability of research findings. In fact, most of the mothers were well-educated and of middle-income status. Therefore, the results of the study may not be representative of population with low socioeconomic status and educational level. Thus, further studies on samples with limited income and education are needed. Finally, despite the sample size of each clinical group was modest, studies involving a relatively small number of samples, especially in clinical population, can produce easily replicable results and be informative in assessment and clinical intervention strategies [73]. Notwithstanding the above limitations, several strengths should also be mentioned. Most studies on feeding disorders in early childhood did not distinguish between different clinical subtypes. Our study, focusing on three specific ARFID sub-types, has added to previous literature new knowledge on associated children’s and mother’s psychopathologic risk and the quality of feeding interactions which, in turn, may be useful to develop the best strategy to treat each disorder. Moreover, no previous study has evaluated the DP of children’s with ARFID. Finally, we used an observational validated tool to study the quality of mother–child interactions during feeding, which has allowed for an objective measure of the child’s emotional and behavioral functioning.

### 4.2. Implications for Practice and Clinic Applications

Our findings suggest that, for early children with ARFID diagnosis, interventions at the level of mother–child feeding interactions are called for. Intervention programs focused on the parent-infant relationship appeared to promote child’s ARFID resolution [74] and video-feedback treatment specifically focused on mother-infant interaction has proven to produce better outcomes compared to treatments focused only on the mother or child [75]. However, some studies have underlined that the lack of father’s involvement may compromise the efficacy of the therapy [76]. Moreover, the use of the coding system of SVIA allows to obtain information on different domains of interactions (child, mother and dyad) and, revealing both difficulties and strengths, may be useful for both assessment and more targeted intervention programs, showing the best entry point for clinical intervention.

## 5. Conclusions

The recent literature in the field of developmental psychopathology framework has underlined that ARFID among early childhood is a serious problem that may be associated with psychological difficulties in child, their mother, as well as with a poor quality of parent-child feeding interactions.

Our findings, considering three specific ARFID subtypes (I/I, SFA, PTFD), have evidenced specific characteristics in the dyads of the three groups: (1), the dyads of I/I group were the most dysfunctional: children reported internalizing and attention problems, as well as emotional and behavioral dysregulation and their mothers reported the highest psychopathologic risk, especially in the depressive area. Moreover, these dyads manifested the poorest quality of feeding exchanges, in all aspects of interactions; (2), the dyads of SFA group, on the other hand, are composed by children with high emotional reactivity, but with a general emotional-behavioral functioning less compromised than other clinical groups. At the same time, their mothers showed the lowest levels of psychopathologic difficulties compared to mothers of other clinical groups and the quality of feeding interactions was under clinical cutoff for all dimensions; finally (3), the dyads of PTFD group, are characterized by children with externalizing problems and the highest levels of emotional and behavioral self-dysregulation (CBCL DP). Their mothers showed the highest obsessive compulsion and anxiety symptoms. These aspects seem reflected in a poor quality of feeding interactions in both children and their mothers, with scores of all scales of SVIA that exceeded the clinical cutoff.

Overall, these findings may have important clinical implication for the development of prevention and treatment programs more targeted and effective.

## Figures and Tables

**Table 1:** Means, standard deviations, Levene, Welch and one-way ANOVA of CBCL 1.5–5 syndrome scales and dysregulation profile on the basis of children diagnoses.

CBCL 1.5–5	Children’s Diagnoses								Levene’s Test *p*-Value	ANOVA and Welch’s ANOVA			
	I/I		SFA		PTFD		NC						
	M	SD	M	SD	M	SD	M	SD		F	df1	df2	*p*
ER ^a^	7,13	2.56	15.16	2.15	6.16	3.18	1.22	1.08	0.000 ***	288.05	3	47	0.000 ***
A/D	13.13	1.98	4.8	2.84	5.20	2.14	2.03	1.37	0.69	119.74	3	96	0.000 ***
SC ^a^	7.34	1.84	4.44	2.46	8	2.97	3.07	1.59	0.02 *	33.56	3	51	0.000 ***
WD ^a^	6.43	2.37	3.52	2.25	5.44	2.23	2.33	1.03	0.02 *	28.12	3	47	0.000 ***
SP ^a^	5.65	1.92	3.56	1.95	4.44	2.43	1.88	1.45	0.03 *	51.52	3	51	0.000 ***
AP	5.47	1.78	2.96	1.90	3.68	1.86	1.74	1.45	0.73	19.55	3	96	0.000 ***
AB ^a^	15.08	4.40	9.96	4.41	27.04	4.60	3.59	1.59	0.000 ***	221.65	3	45	0.000 ***
IntPr ^a^	34.04	5.04	27.92	5.33	24.8	6.17	8.66	2.54	0.006 **	224.83	3	47	0.000 ***
ExPr ^a^	20.56	5.07	13	5.83	30.72	5.24	5.33	2.16	0.002 **	201.09	3	46	0.000 ***
DP ^a^	33.69	5.53	17.72	6.45	35.92	5.28	7.37	2.95	0.04 *	265.51	3	48	0.000 ***

Note. ER = emotional reactivity, A/D = anxious/depressed, WD = withdrawn, SL = sleep problems, AP = attention problems, AB = aggressive behavior, IntPr = internalizing problems, ExPr = externalizing problems, DP = dysregulation profile; I/I = ARFID irritable/impulsive subtype group, SFA = ARFID sensory food aversions subtype group, PTFD = ARFID post-traumatic feeding disorder group, NC = non-clinical group; ANOVA = analyses of variance; * *p* < 0.05, ** *p* < 0.01, *** *p* < 0.001; ^a^ = indicates use of Welch statistic for F values (Levene’s test for homogeneity of variance not meet).

**Table 2:** Multiple comparison post hoc test for the CBCL 1.5–5 syndrome scales and dysregulation profile on the basis of children diagnoses.

	Tukey’s and Dunnett T3 Post Hoc Test											
CBCL 1.5–5	I/I			SFA			PTFD			NC		
	vs. SFA	vs. PTFD	vs. NC	vs. I/I	vs. PTFD	vs. NC	vs. I/I	vs. SFA	vs. NC	vs. I/I	vs. SFA	vs. PTFD
ER ^a^	0.002 **	0.48	0.003 **	0.006 **	0.000 ***	0.008 **	0.48	0.002 **	0.000 ***	0.006 **	0.000 ***	0.000 ***
A/D	0.004 **	0.000 ***	0.000 ***	0.000 ***	0.91	0.000 ***	0.000 ***	0.91	0.000 ***	0.000 ***	0.003 **	0.000 ***
SC ^a^	0.000 ***	0.92	0.005 **	0.002 **	0.000 ***	0.13	0.92	0.000 ***	0.003 **	0.009 **	0.13	0.001 **
WD ^a^	0.000 ***	0.58	0.000 ***	0.007 **	0.02	0.12	0.58	0.02	0.000 ***	0.007 **	0.12	0.000 ***
SP ^a^	0.003 **	0.30	0.000 ***	0.003 **	0.64	0.007 **	0.30	0.64	0.004 **	0.000 ***	0.007 **	0.000 ***
AP ^a^	0.000 ***	0.003 **	0.000 ***	0.000 ***	0.47	0.06	0.003 **	0.47	0.001 **	0.000 ***	0.06	0.001 **
AB ^a^	0.001 **	0.000 ***	0.006 **	0.001 **	0.000 ***	0.005 **	0.000 ***	0.000 ***	0.003 **	0.000 ***	0.000 ***	0.000 ***
IntPr ^a^	0.006 **	0.000 ***	0.005 **	0.000 ***	0.12	0.000 ***	0.000 ***	0.12	0.000 ***	0.008 **	0.000 ***	0.000 ***
ExPr ^a^	0.000 ***	0.000 ***	0.000 ***	0.000 ***	0.000 ***	0.003 **	0.000 ***	0.000 ***	0.000 ***	0.000 ***	0.005 **	0.000 ***
DP ^a^	0.000 ***	0.63	0.000 ***	0.002 **	0.000 ***	0.000 ***	0.63	0.000 ***	0.000 ***	0.000 ***	0.000 ***	0.009 **

Note. ER = emotional reactivity, A/D = anxious/depressed, WD = withdrawn, SL = sleep problems, AP = attention problems, AB = aggressive behavior, IntPr = internalizing problems, ExPr = externalizing problems, DP = dysregulation profile; I/I = ARFID irritable/impulsive subtype group, SFA = ARFID sensory food aversions subtype group, PTFD = ARFID post-traumatic feeding disorder group, NC = non-clinical group; ** *p* < 0.01, *** *p* < 0.001; ^a^ = Multiple comparison were conducted using Dunnett’s T3 test (heterogenic variance).

**Table 3:** Means, standard deviations, Levene and Welch’s ANOVA of the SCL/90-R scales in the four groups.

SCL-90/R	Children’s Diagnoses								Levene’s Test *p*-Value	Welch’s ANOVA			
	I/I		SFA		PTFD		NC						
	M	SD	M	SD	M	SD	M	SD		F	df1	df2	*p*
SOM	1.72	0.40	0.69	0.30	1.2	0.27	0.13	0.12	0.003 **	193.49	3	46	0.000 ***
O–C	1.73	0.43	0.53	0.24	2.90	0.35	0.15	0.18	0.001 **	431.57	3	49	0.000 ***
DEP	2.97	0.74	0.71	0.38	1.06	0.30	0.17	0.17	0.000 ***	139.32	3	47	0.000 ***
I–S	1.75	0.38	0.61	0.29	1.23	0.33	0.18	0.26	0.04 *	110.91	3	51	0.000 ***
ANX	1.84	0.41	0.59	0.28	2.82	0.63	0.21	0.14	0.000 ***	221.66	3	45	0.000 ***
HOS	1.78	0.54	0.52	0.33	1.19	0.41	0.20	0.14	0.000 ***	96.28	3	44	0.000 ***
PHOB	1.81	0.36	0.53	0.29	1.08	0.41	0.16	0.19	0.001 **	139.37	3	49	0.000 ***
PAR	1.71	0.56	0.62	0.34	1.21	0.45	0.16	0.12	0.000 ***	96.51	3	43	0.000 ***
PSY	1.79	0.46	0.47	0.27	1.04	0.36	0.22	0.15	0.000 ***	101.11	3	47	0.000 ***
GSI	1.95	0.22	0.60	0.10	1.53	0.16	0.18	0.05	0.000 ***	855.09	3	46	0.000 ***

Note. SOM = somatization, O–C = obsessive compulsion, DEP = depression, I–S = interpersonal sensitivity, ANX = anxiety, HOS = hostility, PHOB = phobic anxiety, PAR = paranoid ideation, PSY = psychoticism, GSI = global severity index; I/I = ARFID irritable/impulsive subtype group, SFA = ARFID sensory food aversions subtype group, PTFD = ARFID post-traumatic feeding disorder group, NC = non-clinical group. ANOVA = analyses of variance; * *p* < 0.05, ** *p* < 0.01, *** *p* < 0.001.

**Table 4:** Multiple comparison post hoc test for the SCL/90-R scales syndrome scales on the basis of children diagnoses.

	Dunnett T3 Post Hoc Test											
SCL 90/R	I/I			SFA			PTFD			NC		
	vs. SFA	vs. PTFD	vs. NC	vs. I/I	vs. PTFD	vs. NC	vs. I/I	vs. SFA	vs. NC	vs. I/I	vs. SFA	vs. PTFD
SOM	0.004 **	0.003 **	0.001 **	0.000 ***	0.006 **	0.002 **	0.007 **	0.008 **	0.003 **	0.006 **	0.000 ***	0.000 ***
O–C	0.000 ***	0.000 ***	0.000 ***	0.000 ***	0.000 ***	0.000 ***	0.000 ***	0.000 ***	0.000 ***	0.000 ***	0.000 ***	0.000 ***
DEP	0.000 ***	0.003 **	0.005 **	0.000 ***	0.005 **	0.003 **	0.000 ***	0.005 **	0.003 **	0.000 ***	0.000 ***	0.000 ***
I–S	0.001 **	0.000 ***	0.009 **	0.006 **	0.000 ***	0.000 ***	0.004 **	0.000 ***	0.006 **	0.000 ***	0.009 **	0.007 **
ANX	0.000 ***	0.002 **	0.000 ***	0.000 ***	0.000 ***	0.000 ***	0.000 ***	0.000 ***	0.008 **	0.009 **	0.006 **	0.005 **
HOS	0.000 ***	0.001 **	0.000 ***	0.002 **	0.000 ***	0.001 **	0.001 **	0.005 **	0.000 ***	0.000 ***	0.001 **	0.000 ***
PHOB	0.000 ***	0.008 **	0.000 ***	0.009 **	0.000 ***	0.000 ***	0.007 **	0.000 ***	0.000 ***	0.008 **	0.003 **	0.005 **
PAR	0.000 ***	0.001 **	0.000 ***	0.004 **	0.000 ***	0.000 ***	0.001 **	0.000 ***	0.000 ***	0.000 ***	0.005 **	0.005 **
PSY	0.000 ***	0.009 **	0.000 ***	0.000 ***	0.000 ***	0.002 **	0.002 **	0.000 ***	0.007 **	0.000 ***	0.002 **	0.007 **
GSI	0.000 ***	0.000 ***	0.000 ***	0.000 ***	0.000 ***	0.000 ***	0.000 ***	0.000 ***	0.004 **	0.000 ***	0.003 **	0.000 ***

Note. SOM = somatization, O–C = obsessive compulsion, DEP = depression, I–S = interpersonal sensitivity, ANX = anxiety, HOS = hostility, PHOB = phobic anxiety, PAR = paranoid ideation, PSY = psychoticism, GSI = global severity index; I/I= ARFID irritable/impulsive subtype group, SFA = ARFID sensory food aversions subtype group, PTFD = ARFID post-traumatic feeding disorder group, NC = non-clinical group; ** *p* < 0.01, *** *p* < 0.001.

**Table 5:** Means, standard deviations, Levene and Welch’s ANOVA of the SVIA dimensions in the four groups.

SVIA	Children’s Diagnoses								Levene’s Test *p*-Value	Welch’s ANOVA			
	I/I		SFA		PTFD		NC						
	M	SD	M	SD	M	SD	M	SD		F	df1	df2	*p*
ASm	28.21 ^a^	2.12	11.85 ^b^	3.07	21.70 ^c^	2.01	2.58 ^d^	1.11	0.009 **	1212.66	3	48	0.000 ***
IC	25.61 ^a^	1.80	11.93 ^b^	2.33	18.84 ^c^	1.59	2.18 ^d^	0.83	0.001 **	1533.59	3	47	0.000 ***
FRc	15.22 ^a^	1.65	6.33 ^b^	1.69	11.86 ^c^	1.11	1.43 ^d^	0.73	0.01 *	793.24	3	48	0.000 ***
ASd	16.34 ^a^	1.54	6.80 ^b^	1.17	11.95 ^c^	0.97	1.42 ^d^	0.62	0.01 *	1107.64	3	48	0.000 ***
Tot	85.40 ^a^	6.16	36.92 ^b^	6.41	64.36 ^b^	3.80	7.62 ^c^	2.50	0.002 *	1968.86	3	48	0.000 ***

Note. Different letters indicate significant differences between groups. ASm = affective state of the mother, IC = interactional conflict, FRc = food refusal of the child, ASd = affective state of the Dyad, Tot = total score; I/I = ARFID irritable/impulsive subtype group, SFA = ARFID sensory food aversions subtype group, PTFD = ARFID post-traumatic feeding disorder group, NC = non-clinical group; ANOVA = analyses of variance; * *p* < 0.05, ** *p* < 0.01, *** *p* < 0.001.

**Table 6:** Multiple comparison post hoc test for the SVIA scores in the four groups.

	Dunnett T3 Post Hoc Test											
SVIA	I/I			SFA			PTFD			NC		
	vs. SFA	vs. PTFD	vs. NC	vs. I/I	vs. PTFD	vs. NC	vs. I/I	vs. SFA	vs. NC	vs. I/I	vs. SFA	vs. PTFD
ASm	0.005 **	0.008 **	0.006 **	0.000 ***	0.008 **	0.000 ***	0.000 ***	0.000 ***	0.003 **	0.000 ***	0.009 **	0.008 **
IC	0.000 ***	0.006 **	0.000 ***	0.009 **	0.004 **	0.000 ***	0.000 ***	0.004 **	0.005 **	0.006 **	0.000 ***	0.000 ***
FRc	0.002 **	0.004 **	0.005 **	0.000 ***	0.005 **	0.000 ***	0.006 **	0.000***	0.008 **	0.008 **	0.000 ***	0.006 **
AFd	0.009 **	0.000 ***	0.001 **	0.002 **	0.000 ***	0.009 **	0.000 ***	0.001 **	0.000 ***	0.000 ***	0.000 ***	0.004 **
Tot	0.008 **	0.000 ***	0.007 **	0.000 ***	0.000 ***	0.005 **	0.001 **	0.000 ***	0.002 **	0.000 ***	0.003 **	0.000 ***

Note. ASm = affective state of the mother, IC = interactional conflict, FRc = food refusal of the child, ASd = affective state of the Dyad, Tot = total score; I/I = ARFID irritable/impulsive subtype group, SFA = ARFID sensory food aversions subtype group, PTFD = ARFID post-traumatic feeding disorder group, NC = non-clinical group; ** *p* < 0.01; *** *p* < 0.001.
